# Spiking neural network configuration designed for switching between basic forms of movement in a biped robot

**DOI:** 10.1186/1471-2202-16-S1-P104

**Published:** 2015-12-18

**Authors:** Uziel Jaramillo-Avila, Horacio Rostro-González

**Affiliations:** 1 Department of Electronics, Engineering Division, University of Guanajuato, Salamanca, Guanajuato, 36885, México

## 

**Figure 1:**
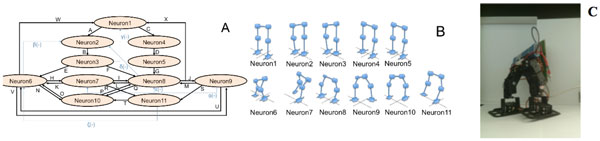
**A. Neuronal network including all the required positions for the 4 basic movements and the 30 synapses required between them**. **B**. Positions of the robot for each one of the 11 neurons. **C. **Biped robot setup.

In this work, we present a neural network design based on a simplified form and highly suitable for hardware implementation of the integrate-and-fire spiking neuron model for achieving locomotion in a biped robot. It is well known that bipedal walking is one of the most complex and common tasks in robots and humanoids and it is also known that this problem is generally tackled by using one type of movement at a time, e.g. forward walking [1]. In this regard, we propose to decompose the movement of a robot with 6 degrees of freedom and to find a series of movements that allow it to take a step forward, a step backwards and make a turn to each side. Thus, a series of 11 commands were successfully achieved, each of them configuring the robot to a specific position that by using them in different sequences, it is possible to perform the movements abovementioned, all starting and ending in a "home position". At the same time, this makes easy switching between them for the movement of the robot in a real environment. Two hypotheses are implicit in this study: on one hand, the movements listed above are equivalent to a spasm or muscle reflex in a biological organism [2]. On the other hand, each one of the commands that makes a simultaneous movement in several motors of the robot is controlled by a spiking neuron, which is attractive because it reflects more closely biological models, and may be more compatible with biomedical applications such as neuroprostheses [3]. To validate our approach, we improved a hardware implementation (on a Spartan 6 FPGA board from Opal Kelly) of the network shown in Figure 1 and tested it successfully on a real biped robot from Lynxmotion.

Once the network topology is configured for one of the movements, a corresponding sequence of neurons is activated as follows:

• Step forward: A-B-E-H-I-J-U-W

• Step backward: A-B-F-L-K-V-M-X

• Turn left: A-B-E-N-P-J-S-T

• Turn right: C-D-G-Q-O-H-R-T
